# VeLoc: Finding Your Car in Indoor Parking Structures

**DOI:** 10.3390/s18051403

**Published:** 2018-05-02

**Authors:** Ruipeng Gao, Fangpu He, Teng Li

**Affiliations:** 1 School of Software Engineering, Beijing Jiaotong University, Beijing 100044, China; 15301039@bjtu.edu.cn; 2 Department of Electrical Engineering and Computer Science, Syracuse University, Syracuse, NY 13244, USA; tli01@syr.edu

**Keywords:** vehicle localization, inertial tracking, virtual landmarks, mobile crowdsensing

## Abstract

While WiFi-based indoor localization is attractive, there are many indoor places without WiFi coverage with a strong demand for localization capability. This paper describes a system and associated algorithms to address the indoor vehicle localization problem without the installation of additional infrastructure. In this paper, we propose VeLoc, which utilizes the sensor data of smartphones in the vehicle together with the floor map of the parking structure to track the vehicle in real time. VeLoc simultaneously harnesses constraints imposed by the map and environment sensing. All these cues are codified into a novel augmented particle filtering framework to estimate the position of the vehicle. Experimental results show that VeLoc performs well when even the initial position and the initial heading direction of the vehicle are completely unknown.

## 1. Introduction

With the rapid growth of the number of personal vehicles and the increase in urban parking space demands, numerous large parking facilities have appeared, most of which are built underground. A driver may spend several minutes finding an available parking space in busy cities. What is worse, the driver is likely to forget the exact position of his car and may find it difficult to search for it in dim and maze-like parking lots. There is need to develop an easy-to-use system without specialized equipment to assist these drivers.

Localizing a vehicle in parking structures is a challenging problem because of the special environment most parking structures have. Although most vehicles are equipped with vehicle-mounted GPS and the mobile devices of the driver also have GPS, the indoor environment (especially underground) makes them impractical to use. The radio frequency (RF) fingerprinting of WiFi signals, which has been a popular approach to indoor localization [1,2,3,4], does not work here due to the poor WiFi coverage in parking lots.

The smartphone is an ideal device to address this problem. If the result of localization is recorded in the smartphone, the driver can use it to find his car when he comes back. In addition, providing drivers with a map of the parking lot and the real-time position of the vehicle can aid them to search for unoccupied parking places, especially in an unfamiliar environment .

In this paper, a system called VeLoc is developed to address the indoor vehicle localization problem. We develop a mobile sensor computing system to help users find their vehicles in parking lots. To use this system and drive safely, the user does not make a phone call but puts the mobile on the platform at the front of the vehicle. While the user is driving around, VeLoc leverages a three-axis accelerometer and a three-axis gyroscope present in the smartphone to track the vehicle and obtain a real-time position. When the vehicle finally stops, our system records the parking position on the map for the user, who may need it to find the vehicle later.

The only external input that VeLoc depends upon is a map of the indoor space of interest, which is necessary not only for the purpose of tracking but also to show the localization result to the users. Unlike common tracking methods, VeLoc does not need the initial state of the vehicle as an input since it is best to minimize the amount of explicit input or other actions from users. Shown in Section 5, VeLoc is robust and performs well even when both the initial position and the initial heading direction are unknown. If other information can be provided for the initial state of the vehicle (for example, in nearly all cases vehicles need to stop at one of the entrances of the parking lots), our system will converge to the true state of the vehicle more quickly.

There are three key ideas behind the automatic estimation of vehicles’ locations in parking lots. We introduce these ideas first, and then describe how they are codified together to address the vehicle localization problem.

(1) *Knowing the starting location, the motion trajectory of a device can, in principle, be computed by integrating inertial sensor measurements over time.* This is called urban dead reckoning. Due to noise in the mobile sensors and the error that occurs during integration, the dead-reckoned trajectories are accurate at the beginning, but diverge from the truth quickly over time. Recalibration is necessary to prevent the accumulation of errors. The observation that smartphones inside vehicles provide simpler motion than for walking people shows possibility to use dead reckoning in the vehicle localization problem.
(2) *The constraints imposed by the map and the regularity of vehicles’ motion filter out the infeasible locations over time and converge on the true location.* Some examples of constraints are that the vehicle cannot move through a wall, or that vehicles in other areas cannot be marked on the map. This information can not only be used as cues for recalibration in dead reckoning but also by itself allows for the localization of a vehicle from inertial sensor measurements. For instance, assuming we have no idea where the vehicle is, inertial sensor measurements will provide a trajectory when the vehicle is moving. While the above information does not, by itself, reveal location, it could when viewed together with a map since there may be only one path on the map that could accommodate the trajectory observed. Thus, at the conclusion of the driving, we can infer that the vehicle’s final location and then trace back to infer the starting location.
(3) *Inertial sensor measurements show particular patterns when the vehicle is turning, coming to a slope, or traveling through “road anomalies", including speed bumps and potholes as well as other rough road conditions [5]*. (We only use particular patterns on inertial sensor measurements to define several landmarks while the indoor environment is rich with ambient signals, like sound, light, magnetic field, temperature, WiFi, 3G, etc. SurroundSense [6] extends this idea and builds a map using several features.) We can define several kinds of landmarks as the positions at which inertial sensors inside vehicles provide measurements with particular patterns. Once those landmarks can be detected automatically, this kind of information can be used to recalibrate dead reckoning. The density of landmarks can be guaranteed since different building structures force vehicles to behave in specific ways and parking lots provide enough “road anomalies”, especially speed bumps.

To combine the above ideas into VeLoc, we represent the uncertainty on the state of vehicles explicitly as probability distributions. We incorporate the uncertainty arising from sensing and the information provided by the map or detected landmarks into a novel Bayes Filtering framework, specifically, the *augmented particle filtering* framework. While particle filtering in the context of localization typically uses particles only to represent the uncertainty in location, we design novel particles that also incorporate the uncertainty in terms of other aspects such as the direction of the smartphone and the velocity of the vehicle. To use landmark information for recalibration, we use pattern recognition and machine learning techniques to find particular patterns and a implement real-time classification algorithm in VeLoc to detect predefined landmarks automatically. The result of detection is used as input to augmented particle filtering algorithm.

The Bayes filtering algorithm possesses two essential steps: the time update and measurement update. Similar to dead reckoning, VeLoc estimates the state of every particle based on the previous state at the time update step. During the measurement update, VeLoc utilizes all the information, as measurements consist of inertial sensor measurements, constraints imposed by the map, and the detected landmark, together with conditional distributions of measurements to estimate the probability of every particle.

Our main contributions may be summarized as follows:

- This is the first system (to our knowledge) to address the real need for localizing vehicles in parking structures where GPS do not work and WiFi signals are not available.
- We identify an opportunity to simultaneously harness constraints imposed by the map and environment sensing for localization. Our approach does not require calibration nor the installation of additional infrastructure.
- VeLoc overcomes the error accumulation problem. We show experimentally that VeLoc achieves a localization error of 3~5 m in an underground parking structure.

The rest of the paper is organized as follows. Section 2 presents the background and related work, followed by system architecture in Section 3. Design details are presented in Section 4, while results is discussed in Section 5. Section 6 concludes this paper and discusses future work.

## 2. Background and Related Work

VeLoc draws on prior work in multiple areas, including dead reckoning, robotic navigation, and road surface monitoring.

### 2.1. Dead Reckoning

Dead reckoning using inertial sensors is a well-studied research area. Typical sensors used in such domains are of high-quality but expensive. Utilizing noisy smartphone sensors in indoor environments [7] is attractive since consumer mobile devices such as smartphones are increasingly being equipped with sensors such as accelerometers, gyroscopes, magnetometers, and barometers. However, directly applying the idea to human-scale environments is non-trivial since several factors cause fluctuations in acceleration, resulting in erroneous displacements.

Many methods have attempted to address the accumulation of error. Foot-mounted sensors are able to reduce the error [8,9], but the accumulation of error still remains and this method is not in accordance with the typical placement of devices such as smartphones. Outdoor localization schemes like CompAcc [10] have triggered periodic GPS measurements to recalibrate the user’s location. UnLoc [11] presents an option to replace the GPS with virtual indoor landmarks that are created using existing sensing modalities. In the area of robotics, methods based on map of the place of interest implement high-accuracy localization [12,13]. Mobile computing uses this idea to address the indoor localization problem [14], and characterizations of the smartphone’s inertial sensors are well investigated for vehicle tracking [15,16,17].

To prevent the error from being accumulated, VeLoc simultaneously harnesses constraints imposed by the map and environment sensing. The only external input that VeLoc depends on is a map of the indoor space of interest. Since the map of a place does not change for several months or years, no calibration is needed in VeLoc. In addition, the need for special-purpose hardware and infrastructure is avoided to make VeLoc more practical for real use.

### 2.2. Robotic Navigation

A highly popular and successful technique in robotics, called SLAM, allows the robot to acquire a map of its environment while simultaneously localizing itself relative to this map [18]. Recently, WiFi-SLAM [19] proposed the usage of the WiFi signal for SLAM. Unlike SLAM, VeLoc assumes the availability of a map and the problem to be addressed is mobile robot localization. Mobile robot localization, which is often called position estimation or position tracking, is the problem of determining the pose of a robot relative to a given map of the environment [20].

The early work on mobile robot localization problem used Kalman filters, which are thought to be the earliest tractable implementations of the Bayes filter for continuous spaces. Subsequent work has been based on Markov localization, which is a better match in practice since it allows the robot’s position to be modeled as multi-modal and non-Gaussian probability density functions. Of particular interest to us is the Monte Carlo localization (MCL), or particle filtering-based approach [21,22]. Instead of representing the distribution by a parametric form, particle filters represent a distribution by a set of samples drawn from this distribution. Those particles are then evolved based on the action model and the measurements [20].

However, robot localization typically depends on using explicit environment sensors, such as laser range finders and cameras. Moreover, the rotation of the robot wheels offers a precise computation of displacement. Unlike robot localization, VeLoc uses smartphone sensors to compute the displacement and direction of vehicles, and landmarks are essentially virtual landmarks, as described next.

### 2.3. Virtual Landmarks

In the mobile robot localization problem, robots equipped with sensors such as laser-based ranging and cameras are assumed to be exploring the space of interest. The space is assumed to have landmarks, which are typically artificially inserted (e.g., barcodes pasted on walls or a particular pattern painted on the ceiling). However, in the area of indoor localization, smartphones, not robots, are carried around in the space of interest. We cannot depend on additional sensors since these are either not present in typical consumer devices (e.g., laser ranging) or even if present are not amenable to use (e.g., smartphone cameras). Thus we need resort to virtual landmarks which are essentially ambient signatures or recognized activities.

Ambient signatures and activity recognition have been utilized [6,23], and UnLoc [11] is believed to be the first to apply virtual landmarks for dead reckoning. $P^{2}$ [5] uses mobile sensor measurements to detect road anomalies for the purpose of road surface monitoring. VeTrack [24] and Jigsaw [25] identify visual landmarks and construct maps of shopping malls with parking structures. In VeLoc, we use sensor measurements to detect all kinds of road anomaly and turnings as landmarks. In addition, sensor measurements also provide cues for estimating the state of vehicles. For instance, different patterns between a static vehicle and a moving one can be viewed as a measurement of the velocity of the vehicle.

## 3. System Architecture

**Figure 1:**
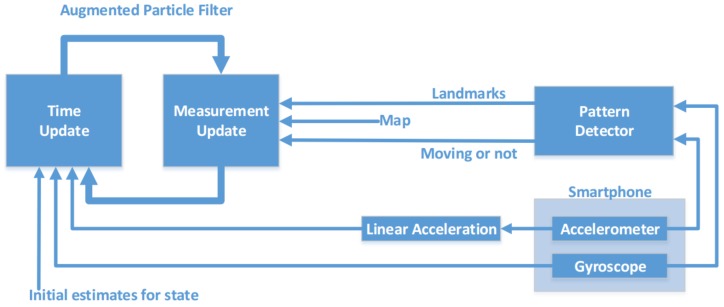
Indoor vehicle localization architecture.

In this section, we describe the software architecture of VeLoc and Figure 1 depicts a pictorial overview of the architecture. There are two key components in VeLoc: the pattern detector (PD) and the augmented particle filter (APF). The PD uses pattern recognition techniques to analyze the inertial sensor measurements. The result of the PD consists of detected landmarks whether the vehicle is moving or not. The output of the PD together with the map and inertial sensor measurements is used at the measurement update step of the APF. Note that raw accelerometer measurements are replaced with linear acceleration, which excludes the effect of the Earth’s gravity. The APF performs time update and measurement update recursively to estimate the state of the vehicle over time.

**The Pattern Detector:** The PD uses the accelerometer and gyroscope data to perform two key functions. The first function reliably determines whether the vehicle is moving. The fundamental observation behind this is that if the vehicle is moving there exists a high frequency vibration in the output of accelerometer regardless of the way the vehicle moves. Second, the PD views measurements as signals over time and analyzes subsection of those signals for a specified time duration to determine whether a landmark is encountered. Landmarks used in VeLoc includes turnings and road anomalies.

**The Augmented Particle Filter:** The key function of the APF is to track the probability distribution of a vehicle’s state as it moves. Since sensors on a smartphone only provide measurements for acceleration but not velocity, a natural computation method is to double-integrate acceleration as ∫∫a(t)dtdt, where the first integration computes speed and the second computes displacement. Unfortunately, several factors cause fluctuations in acceleration, resulting in erroneous displacements. One solution to address this problem is to view velocity as one dimension of the state and simultaneously estimate velocity with the location and heading direction. VeLoc maintains a four-dimensional joint probability distribution function in the form of a particle filter and estimates all these values as the vehicle moves on the floor.

## 4. Design Details

This section describes the algorithms and engineering details underlying VeLoc.

**Figure 2:**
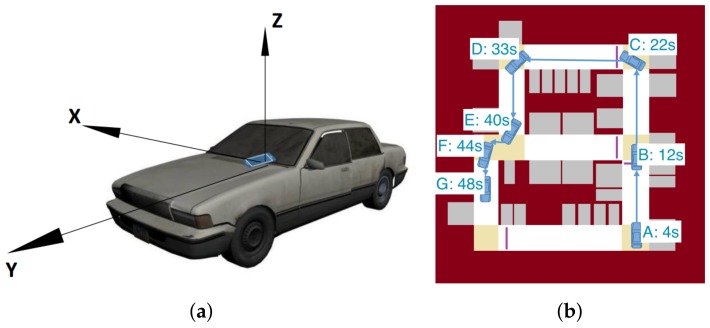
The vehicle coordinate system and the map of an example vehicle route. Seven key points are demonstrated with time stamps. The vehicle starts at A and stops at G. The timer starts before the vehicle starts and its time is recorded at every key point. (**a**) Vehicle coordinate system; (**b**) Map of the example scenario shown with the route.

To use VeLoc, users need to put their smartphones on the platform at the front of the vehicle. Also, the smartphone needs to be facing towards the front. Thus, we can define the coordinate system of the vehicle in the same way as the smartphone. Figure 2a shows how the coordinate system of the smartphone and the coordinate system of the vehicle are related.

### 4.1. Pattern Detection

**Figure 3:**
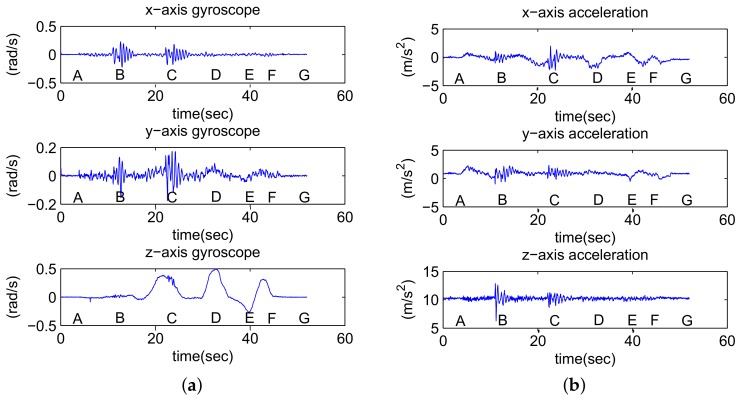
Sensor data recorded during the example drive. (**a**) Gyroscope; (**b**) Acceleration.

The intuition behind our algorithm is that both anomalous road conditions and whether the vehicle is moving are reflected in features of the acceleration data. For the sake of clarity, we will illustrate this part with an example scenario. Figure 2b shows the map of an underground parking lot with the route drawn on it. Seven key points, i.e., A to G, are demonstrated with time stamps. Figure 3 shows the sensor data while the vehicle is moving along the route.

We will discuss the features used in different tasks first and then use a machine learning method to optimize all the parameters in our model.

#### 4.1.1. Moving Detection

We can distinguish the static state from a state of movement with constant speed, in principle, if the floor is absolutely flat. However, in practice, the floor of the parking lot cannot be that flat at all, which causes vibration in sensor data while the car is moving. Based on this intuition, VeLoc calculates the following feature of a signal s(t):

(1) $$E\left(s,t\right)=\int_{t-\delta }^{t}s{\left(x\right)}^{2}dx$$

and the discrete version (the first item) with standardization (the second item):

(2) $$E\left(s,t\right)=\sum_{i=t-k+1}^{t}s{\left(i\right)}^{2}-\frac{1}{k}{\left\{\sum_{i=t-k+1}^{t}s(i)\right\}}^{2}$$

where both δ and *k* describe the window size. In our implementation, k=100, corresponding to δ=2s since sensor data is collected every 0.02s in VeLoc (the default sampling rate for inertial sensors in iOS). The feature used in moving detection is defined as:

(3) $$F_{1}\left(t\right):=w_{11}E\left(acc_{x},t\right)+w_{12}E\left(acc_{y},t\right)+w_{13}E\left(acc_{z},t\right)$$

where $w_{11},w_{12},w_{13}$ are weights summing up to 1.

The vehicle is regarded as static at time *t* if and only if the following inequality is satisfied:

(4) $$F_{1}\left(t\right)<thres_{1}$$

where $thres_{1}$ is a threshold.

**Figure 4:**
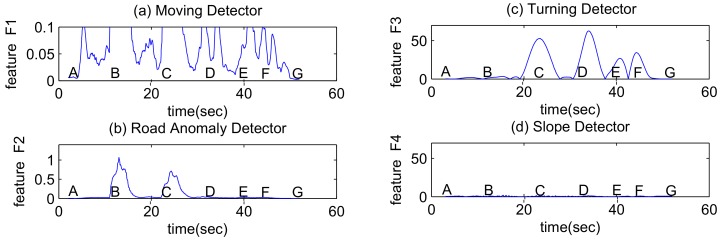
Features calculated for different detectors.

During the example drive, the feature $F_{1}$ calculated for moving detection is shown in Figure 4a. Note that only two key points, A(4s) and G(48s), show relatively small values.

#### 4.1.2. Road Anomaly Detection

When a vehicle encounters speed bumps, potholes, or other rough road conditions, there are high-energy events in both acceleration signals and gyroscope signals. Acceleration along the z-axis best characterizes this anomaly since vibrating up and down changes the acceleration along this direction. Gyroscope signals along the x- and y-axes can also be used to detect road anomalies because these signals are always close to zero unless there is vibration caused by a road anomaly.

We use the definition of feature E in Section 4.1.1 to define the feature for road anomaly detection as follows:

(5) $$F_{2}\left(t\right):=w_{21}E\left(acc_{z},t\right)+w_{22}\frac{E(gyr_{x},t)}{b}+w_{23}\frac{E(gyr_{y},t)}{b}$$

where $w_{11},w_{12},w_{13}$ are weights summing up to 1. *b* is used to balance the difference in scale between two kinds of data. In VeLoc, we set $b={\left(0.1\right)}^{2}=0.01$.

The road anomaly is detected at time *t* if and only if the following inequality is satisfied:

(6) $$F_{2}\left(t\right)>thres_{2}$$

where $thres_{2}$ is a threshold.

During the example drive, the feature $F_{2}$ calculated for road anomaly detection is shown in Figure 4b. Note there are two responses corresponding to two speed bumps shown in the Figure 3, and the activities occur after key points B (12s) and C (22s). The magnitude of the response also depends on the velocity of the vehicle. At high speeds, even small road anomalies can create high peak acceleration readings. This can be used to explain why the second speed bump causes a lower peak in the $F_{2}$ feature since the vehicle turns earlier and the velocity is relatively low.

#### 4.1.3. Turning Detection and Slope Detection

Both turnings and slopes can be detected by gyroscope signals since rotational velocities are measured. Turnings result in rotational velocity along the z-axis, while slopes result in rotational velocity along the x-axis. Based on this intuition, VeLoc calculates the following feature of a signal s(t):

(7) $$C\left(s,t\right)=|\int_{t-\delta }^{t}s(x)dx|$$

with discrete version:

(8) $$C\left(s,t\right)=|\sum_{i=t-k+1}^{t}s(i)|$$

where both δ and *k* describe the window size. In our implementation, k=150, corresponding to δ=3s since it may take longer time to travel through a turn.

A turn is detected at time *t* if and only if the following inequality is satisfied:

(9) $$F_{3}\left(t\right):=C\left(gyro_{z},t\right)>thres_{3}$$

where $thres_{3}$ is a threshold.

A slope is detected at time *t* if and only if the following inequality is satisfied:

(10) $$F_{4}\left(t\right):=C\left(gyro_{x},t\right)>thres_{4}$$

where $thres_{1}$ is a threshold.

During the example drive, the feature $F_{3}$ calculated for turn detection is shown in Figure 4c while feature $F_{4}$ calculated for slope detection is shown in Figure 4d. Note there are four responses corresponding to four turnings shown in the Figure 2. The vehicle turns around key points C (22s), D (33s), E (40s), F (44s). Since there is no slope, feature $F_{4}$ always has a very small response.

The magnitude of the response depends on the degree of the angle that the vehicle turns. For instance, the vehicle does not perform a full 90-degree turn at the two consecutive turnings at E, F, which correspond to two lower peaks.

#### 4.1.4. Training The Detectors

**Figure 5:**
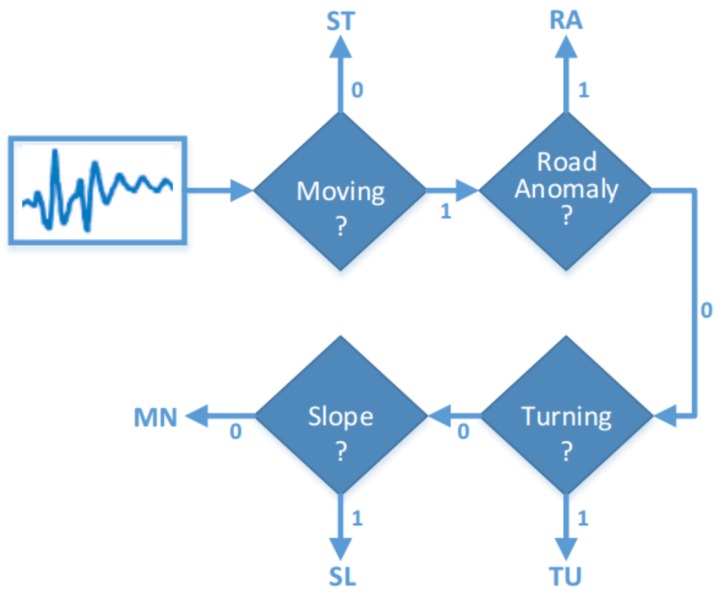
Classifier composed of four pattern detectors.

At time *t*, VeLoc learns to assign a label to indicate the state of the vehicle. The classifier shown in Figure 5 is composed of pattern detectors described above. The labels can take the five different values shown in Table 1.

The detectors described above are controlled through tuning parameters **thres** = $[thres_{1},thres_{2},\;thres_{3},thres_{4}]$ and **w** $=[w_{11},w_{12},w_{13},w_{21},w_{22},w_{23}]$, which control the thresholds and weights for predicting labels.

For each set of parameter values **thres** and **w**, we compute loss function loss(**thres**,**w**) as follows:

(11) $$loss\left(\mathrm{thres},w\right)=\sum_{t\in T}\frac{\delta_{\mathrm{thres},w}\left(t\right)}{\left|T\right|}$$

where

(12) $$\delta_{\mathrm{thres},w}\left(t\right)=\left\{\begin{array}{cc} 0, & \mathrm{predicted}\;\mathrm{value}\;\mathrm{equals}\;\mathrm{labeled}\;\mathrm{value};\\ 1, & \mathrm{else}. \end{array}\right.$$

The final parameter set is chosen to minimize the loss function:

(13) $$\begin{array}{cc} \underset{\mathrm{thres},w}{\mathrm{minimize}} & loss(\mathrm{thres},w)\\ \mathrm{subject}\;\mathrm{to} & w_{11}+w_{12}+w_{13}=1,\\ & w_{21}+w_{22}+w_{23}=1. \end{array}$$

The values of these parameters are computed using a combined exhaustive search method. Specifically, we manually set the reasonable searching range and step for each parameter after careful observations of the corresponding sensory data.

### 4.2. Augmented Particle Filter

In this section we describe how VeLoc uses the augmented particle filter (APF) to track the vehicles’ paths as they move in a parking lot. We will first introduce the APF algorithm and then describe the time update and the measurement update, which are the two essential steps in all Bayes filter-based algorithms.

The particle filter is an alternative nonparametric implementation of the Bayes filter. The key idea of the particle filter is to represent a distribution by a set of samples drawn from this distribution. In particle filters, the samples of a posterior distribution are called *particles* and are denoted as:

$$X_{t}:=x_{t}^{\left[1\right]},x_{t}^{\left[2\right]},\ldots ,x_{t}^{\left[M\right]}$$

Each particle $x_{t}^{\left[m\right]}$(with 1≤m≤M) is a concrete instantiation of the state at time *t*, that is, a hypothesis as to what the true world state may be at time *t*. Here *M* denotes the number of particles in the particle set $X_{t}$.

*Augmented particles* are novel particles that also incorporate the uncertainty in other aspects such as the direction of the smartphone and the velocity of the vehicle.

Just like all other Bayes filter algorithms, the APF algorithm constructs the distribution at time *t* recursively from the distribution one time step earlier. Since beliefs are represented by sets of particles, this means that the APF constructs the particle set $X_{t}$ recursively from the set $X_{t-1}$.

The following is the pseudocode for APF algorithm (Algorithm 1) in VeLoc, where $u_{t}$ and $z_{t}$ are control and measurement, respectively, at time *t*, and ~ denotes that a variable is subject to a certain probability distribution.
**Algorithm 1**
$Augmented\_Particle\_Filter\left(X_{t-1},u_{t},z_{t}\right)$.1:${\bar{X}}_{t}=X_{t}=\emptyset$2:**for** m=1 to *M* **do**3: sample $x_{t}^{\left[m\right]}∼p\left(x_{t}|u_{t},x_{t-1}^{\left[m\right]}\right)$4: ${\bar{X}}_{t}={\bar{X}}_{t}\cup \left\{x_{t}^{\left[m\right]}\right\}$5:**end for** 6:**for** m=1 to *M* **do**7: $w_{t}^{\left[m\right]}=p\left(z_{t}|x_{t}^{\left[m\right]}\right)$8:**end for** 9:**for** m=1 to *M* **do**10: draw $x_{t}^{\left[i\right]}$ from ${\bar{X}}_{t}$ with probability ∝ $w_{t}^{\left[i\right]}$11: $X_{t}=X_{t}\cup \left\{x_{t}^{\left[i\right]}\right\}$12:**end for** 13:**return** $X_{t}$


*Time update*, which is also known as *control update*, is shown in Lines 2 through 5, while *measurement update* is shown in Lines 6 through 12.

During the time update, the APF generates a hypothetical state $x_{t}^{\left[m\right]}$ for time t based on the particle $x_{t-1}^{\left[m\right]}$ and the control $u_{t}$. The resulting sample is indexed by *m*, indicating that it is generated from the *m*-th particle in $X_{t-1}$. This step involves sampling from the transition distribution $p(x_{t}|u_{t},x_{t-1}^{\left[m\right]})$ which is not always possible for arbitrary distributions.

During the measurement update, the APF first calculates for each particle $x_{t}^{\left[m\right]}$ the so-called importance weights, denoted $w_{t}^{\left[m\right]}$. Importance weights are used to incorporate the measurement $z_{t}$ into the particle set. The importance weight, thus, is the probability of the measurement $z_{t}$ under the particle $x_{t}^{\left[m\right]}$, that is, $w_{t}^{\left[m\right]}\propto p\left(z_{t}|x_{t}^{\left[m\right]}\right)$. The real “trick” of the particle filter algorithm occurs in Lines 9 through 12, which implement what is known as *resampling* or *importance resampling*. The algorithm draws with *M* replacement particles from the temporary set ${\bar{X}}_{t}$ . The probability of drawing each particle is given by its importance weight. By incorporating the importance weights into the resampling process, the distribution of the particles changes over time.

In the rest of this section, we describe the transition distribution, importance weights, and resampling method in VeLoc.

#### 4.2.1. Transition Distribution

**Figure 6:**
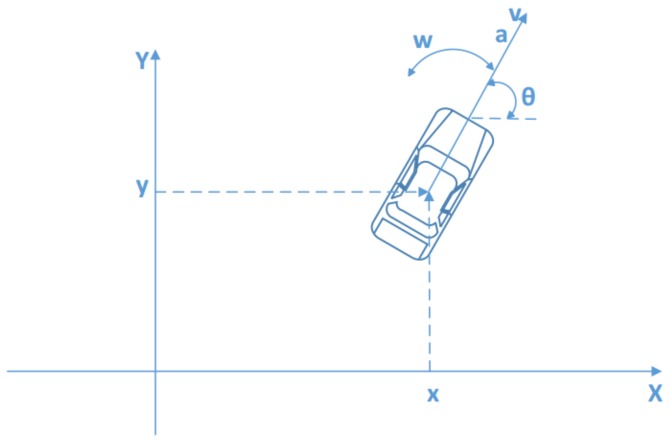
State and control.

To define the transition distribution in VeLoc, we need first introduce the state of a particle and the control. The state of a particle is a four-dimensional vector defined as follows:

〈x,y,θ,v〉

where x,y are position of the vehicle, θ is the heading direction of the vehicle, and *v* is the velocity along the y-axis of the vehicle shown in Figure 6. The control is defined as follows:

〈w,a〉

where *w* is the rotational velocity along the z-axis of the vehicle and *a* is the acceleration along the y-axis of the vehicle shown in Figure 6.

We use the four-dimensional Gaussian distribution

(14) $$N\left(x_{t}|\mu_{t},\Sigma_{t}\right)=\frac{1}{4\pi^{2}}\frac{1}{|\Sigma_{t}{|}^{\frac{1}{2}}}e^{-\frac{1}{2}{\left(x_{t}-\mu_{t}\right)}^{T}\Sigma_{t}^{-1}\left(x_{t}-\mu_{t}\right)}$$

to represent the transition distribution in VeLoc.

$\mu_{t}$ is defined as follows:

(15) $$\mu_{t}=G\left(x_{t-1}\right)+H\left(z_{t}\right)$$

where

(16) $$G\left(x_{t-1}\right)=G\left(\left(\begin{array}{c} x_{t-1}\\ y_{t-1}\\ \theta_{t-1}\\ v_{t-1} \end{array}\right)\right)=\left(\begin{array}{c} x_{t-1}+v_{t-1}\Delta t\cdot \cos\theta_{t-1}\\ y_{t-1}+v_{t-1}\Delta t\cdot \sin\theta_{t-1}\\ \theta_{t-1}\\ v_{t-1} \end{array}\right)$$

and

(17) $$H\left(z_{t}\right)=H\left(\left(\begin{array}{c} w_{t}\\ a_{t} \end{array}\right)\right)=\left(\begin{array}{c} 0\\ 0\\ w_{t}\Delta t\\ a_{t}\Delta t \end{array}\right)$$

$\Sigma_{t}$ is defined as follows:

(18) $$\Sigma_{t}=\left(\begin{array}{cccc} \sigma_{x}^{2} & 0 & 0 & 0\\ 0 & \sigma_{y}^{2} & 0 & 0\\ 0 & 0 & \sigma_{\theta }^{2} & 0\\ 0 & 0 & 0 & \sigma_{v}^{2} \end{array}\right)$$

where all $\sigma^{2}$ describe measurement noise.

#### 4.2.2. Importance Weights

We define the measurement $z_{t}$ in VeLoc as follows:

〈roadanomaly,turning,slope,moving,reachability〉

Every element can take 0 or 1 so that $z_{t}\in {\left\{0,1\right\}}^{5}$. The first three elements are outputs of PD, indicating whether a landmark is encountered. The element moving is also one output of PD, indicating whether the vehicle is moving. The last element reachability indicates whether the vehicle can reach the current position and obviously it is always 1.

To calculate the probability $p(z_{t}|x_{t}^{\left[m\right]})$, we assume that elements of $z_{t}$ are independent so that:

(19) $$p\left(z_{t}|x_{t}^{\left[m\right]}\right)=\prod_{i=1}^{5}p(z_{ti}|x_{t}^{\left[m\right]})$$

As described before, $w_{t}^{\left[m\right]}\propto p\left(z_{t}|x_{t}^{\left[m\right]}\right)$. We can also calculate $w_{t}^{\left[m\right]}$ as:

(20) $$w_{t}^{\left[m\right]}:=\prod_{i=1}^{5}w_{ti}^{\left[m\right]}$$

where for 1≤i≤5

(21) $$w_{ti}^{\left[m\right]}\propto p\left(z_{ti}|x_{t}^{\left[m\right]}\right)$$

In the rest of this part we will introduce the way to calculate $w_{ti}^{\left[m\right]}$ for 1≤i≤5.

$w_{t1}^{\left[m\right]}$ for roadanomaly: Since all the road anomalies are noted on the map, we can calculate for every position (x,y) the closest distance to any road anomalies $dist_{RA}\left(x,y\right)$. $w_{t1}^{\left[m\right]}$ is defined as follows:

(22) $$w_{t1}^{\left[m\right]}=\left\{\begin{array}{cc} 1, & road\;anomaly=0;\\ N(dist_{RA}\left(x_{t1}^{\left[m\right]},x_{t2}^{\left[m\right]}\right)|0,\sigma_{RA}^{2}), & road\;anomaly=1. \end{array}\right.$$

when a road anomaly is detected, a particle which is closer to a road anomaly noted on the map will have a bigger importance weight.

Similarly, $w_{t2}^{\left[m\right]}$ and $w_{t2}^{\left[m\right]}$ can be defined as follows:

(23) $$w_{t2}^{\left[m\right]}=\left\{\begin{array}{cc} 1, & turning=0;\\ N(dist_{TU}\left(x_{t1}^{\left[m\right]},x_{t2}^{\left[m\right]}\right)|0,\sigma_{TU}^{2}), & turning=1. \end{array}\right.$$

(24) $$w_{t3}^{\left[m\right]}=\left\{\begin{array}{cc} 1, & slope=0;\\ N(dist_{SL}\left(x_{t1}^{\left[m\right]},x_{t2}^{\left[m\right]}\right)|0,\sigma_{SL}^{2}), & slope=1. \end{array}\right.$$

$w_{t4}^{\left[m\right]}$ for moving: When the vehicle is detected to be static, a particle with velocity close to 0 should have a large importance weight. Thus, we define $w_{t4}^{\left[m\right]}$ as follows:

(25) $$w_{t4}^{\left[m\right]}=\left\{\begin{array}{cc} N(x_{t4}^{\left[m\right]}|0,\sigma_{MV}^{2}), & moving=0;\\ 1, & moving=1. \end{array}\right.$$

$w_{t5}^{\left[m\right]}$ for reachability: To use the constraints imposed by the map, we give a 0 importance weight to those particles whose positions are noted not able to reach on the map. Assume that R(x,y) is the accessible matrix provided by the map to indicate whether the position (x,y) is able to be reached. Thus, we define $w_{t5}^{\left[m\right]}$ as follows:

(26) $$w_{t5}^{\left[m\right]}=\left\{\begin{array}{c} 0,\;R(x_{t1}^{\left[m\right]},x_{t2}^{\left[m\right]})=0;\\ 1,\;R(x_{t1}^{\left[m\right]},x_{t2}^{\left[m\right]})=1. \end{array}\right.$$

#### 4.2.3. Resampling Method

VeLoc uses the low variance sampler [20] to fulfill the resampling task. It is worth mentioning that since resampling is most time-consuming part of the algorithm, VeLoc does not perform resampling after every update. VeLoc maintains for each particle an importance weight that is initialized by 1 after resampling and updated multiplicatively untill next resampling. In Veloc, resampling is performed every 10 updates.

## 5. Results

**Figure 7:**
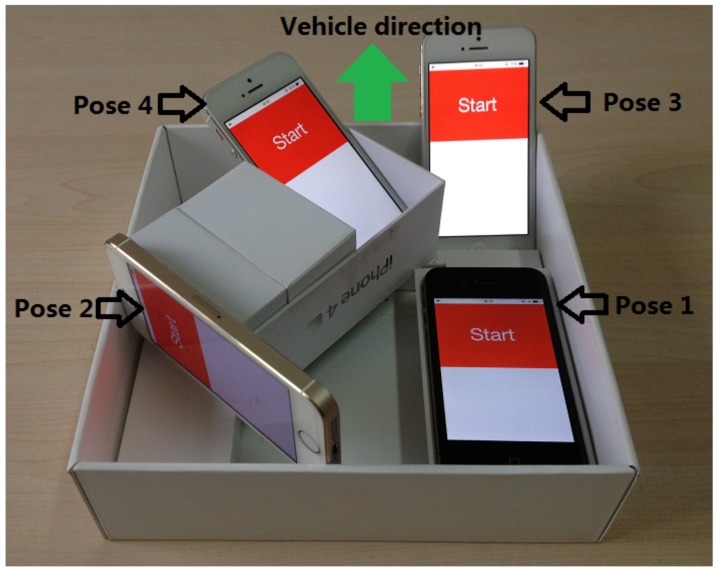
A platform is with four iPhones.

**Methodology**: we implement VeLoc on iOS, and our code includes C++ for algorithms and Objective C for sensor and GUI operations. During experiments, we use smartphones to collect motion sensor readings on a vehicle in an underground parking structure, and its size is 80 m × 90 m. We conduct 20 vehicle traces in the parking structure with four iPhones with different poses to simultaneously collect inertial sensor data during the drive. We design a platform to fix four iPhones with different poses as shown in Figure 7. The platform is placed flat inside the vehicle with a forward direction. To evaluate our system’s robustness, we invited three volunteers to drive their own cars in one parking structure. The cars had been purchased at around 10,000, 20,000, and 30,000 dollars, respectively.

**Figure 8:**
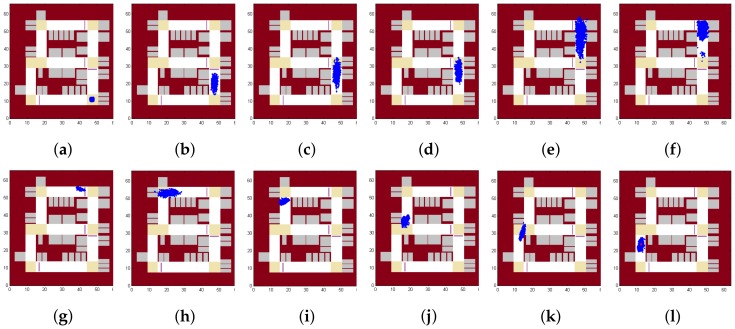
Particles over time.

We first show results in Figure 8, which shows the positions of all the particles as time goes by. In this case, the initial state is informed as being somewhere near the entrance of the parking lot. As shown in Figure 8a, all the particles are initialized around the entrance. Figure 8b shows that particles diverge due to the noise in measurement. Figure 8c,d show how speed bumps could make the particles closer. Figure 8e,f show how turns could make the particles closer. Figure 8g shows all the particles after another turn. Figure 8h,i show the ability to estimate the velocity of the vehicle, as particles with too high or too low velocity will cause the particles to reach a wall noted on the map. Figure 8j,k show how particles pass through two consecutive turns. Figure 8l shows the final position of all the positions and the average position is shown with a red point. Compared with the route shown in Figure 2, VeLoc obtains a very accurate result in the localization problem.

**Figure 9:**
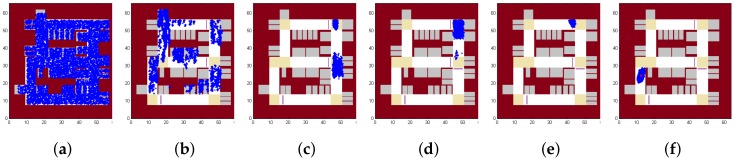
Particles over time without the initial position.

Figure 9 shows the results if the initial position is unknown but the initial heading direction is somehow known (e.g, using compass and the regularity of heading directions of a vehicle in the parking lot). As shown in Figure 9a, particles are initialized everywhere in the parking lot. While the vehicle is moving, constraints imposed by the map filter out some particles as shown in Figure 9b. Figure 9c shows how particles converge quickly when a landmark is detected. Figure 9d,e show the effect of velocity estimation. Figure 9e looks similar to Figure 8g, meaning that VeLoc succeeds in estimating the state of the vehicle without the initial position. We can also trace back to determine the initial position of the vehicle, which is not given at the beginning. Figure 9f shows the final position of all the positions and the average position is shown with a red point. Compared with the route shown in Figure 2, VeLoc gets a very accurate result in the localization problem, without an initial position.

**Figure 10:**
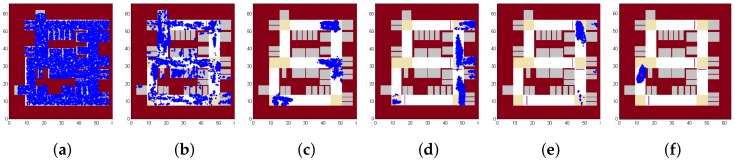
Particles over time without the initial position and the initial heading direction.

Figure 10 shows the results if both the initial position and initial heading direction are unknown. VeLoc is still able to estimate the true state of the vehicle but it may take a little longer to converge. As shown in Figure 10a, particles are initialized everywhere in the parking lot. Constraints imposed by the map filter out some particles but the appearance is still poor, as shown in Figure 10b. Figure 10c shows how particles converge quickly when a landmark is detected. However, compared with Figure 9c, particles converge in more places since the heading direction is unknown. Figure 10d shows that constraints imposed by map filter out some other particles. Figure 10e shows that particles converge after a second landmark detected. Figure 10f shows the final position of all the positions, and the average position is shown with a red point. Compared with the route shown in Figure 2, VeLoc obtains a very accurate result in the localization problem when both the initial position and the initial heading direction are unknown.

**Figure 11:**
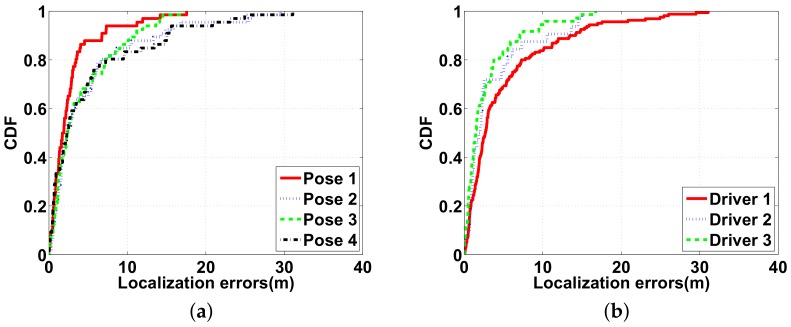
Vehicle localization errors in different scenarios: (**a**) four different poses; (**b**) three different cars and drivers in one parking structure.

Figure 11 shows the vehicle localization accuracy in different scenarios. The 90-percentile localization error is around 10 m for all four poses, and the maximum errors are about 30 m, as shown in Figure 11a. Additionally, as Figure 11b shows, different drivers with their own cars achieve a localization accuracy of around 10 m at the 90th percentile.

## 6. Conclusions and Future Work

In this paper we describe how VeLoc can track the vehicle’s movements and estimate the final parking location using the smartphone’s inertial sensor data only. It does not depend on GPS nor WiFi signals, which may be unavailable in environments such as underground parking lots. It does not require additional sensors to determine the environment. VeLoc first detects landmarks such as speed bumps, turns, and slopes, and combines them with the map information to estimate the vehicle’s location using a probabilistic model. Experiments have shown that VeLoc can track the parking locations to within four parking spaces, which is sufficient for the driver to trigger a honk using the car key.

Currently VeLoc depends on accurate parking structure maps to reduce the uncertainty in vehicle location. Since such maps are not always available, we plan to study how to obtain the map information and track the vehicle when only incomplete and/or inaccurate maps are available. This will further extend VeLoc’s capabilities in the real world.

## Figures and Tables

**Table 1:** Meanings and conditions of labels.

Labels	Meanings	Conditions
IM	Static	Moving detector returns 0
RA	Road anomaly	Anomaly detector returns 1
TU	Turnings	Turning detector returns 1
SL	Slopes	Slope detector returns 1
MN	Moving normally	Else
